# Intra-tumor and peritumoral radiomics and deep learning based on ultrasound for differentiating fibroadenoma and phyllodes tumor: a multicenter study

**DOI:** 10.3389/fonc.2025.1668793

**Published:** 2025-10-23

**Authors:** Guoxiu Lu, Ronghui Tian, Wei Yang, Dongmei Liu, Wenjing Chen, Jingjing Liang, Qi Peng, Shanhu Hao, Guoxu Zhang

**Affiliations:** ^1^ Department of Nuclear Medicine, General Hospital of Northern Theater Command, Shenyang, Liaoning, China; ^2^ School of Software, Shenyang University of Technology, Shenyang, Liaoning, China; ^3^ Department of Radiology, Cancer Hospital of China Medical University, Liaoning Cancer Hospital and Institute, Shenyang, Liaoning, China; ^4^ Department of Ultrasound, Beijing Shijitan Hospital, Capital Medical University, Beijing, China; ^5^ Department of Research and Development, United Imaging Intelligence (Beijing) Co., Ltd., Beijing, China

**Keywords:** fibroadenoma, phyllodes tumor, intratumoral, peritumoral, radiomics, deep learning, ultrasound

## Abstract

**Purpose:**

To develop and validate an integrated intra-tumoral (ITR) and peritumoral (PTR) radiomics-deep learning model based on ultrasound (US) imaging for accurately differentiating fibroadenomas (FA) from phyllodes tumors (PT) and further classifying PT into benign, borderline, and malignant subtypes.

**Methods:**

This multicenter retrospective study enrolled 300 patients (141 FA, 159 PT) from three institutions. US images were analyzed using manual segmentation of ITR and PTR (4mm, 8mm, 12mm, 16mm expansions). A total of 114 radiomics features were extracted per region using PyRadiomics. Five deep learning models (CNN, MLP, ViT, GAN, RNN) and six machine learning classifiers were evaluated. Optimal features were selected via LASSO and Boruta algorithms. Integrated models combining radiomics (ITR ± PTR) with clinical factors (diameter, Bi-RADS) were developed. Performance was assessed using AUC, accuracy, sensitivity, specificity, F1-score, and biopsy reduction rate. Internal validation used a 7:3 random split stratified by center and pathology. External validation was performed on a per-center hold-out basis.

**Results:**

The combined model (ITR + 8mm PTR + clinical) achieved the highest performance for FA/PT differentiation (AUC: 0.960; accuracy: 96.0%; sensitivity: 96.0%; specificity: 94.5%). For PT subtyping (benign/borderline/malignant), the model attained an AUC of 0.874 (accuracy: 77.2%). The integrated model significantly reduced unnecessary biopsy rates by 11.7% overall (18.1% for PT cases). Peritumoral analysis (8mm PTR) contributed critically to model performance, likely capturing stromal interactions at the tumor periphery.

**Conclusion:**

Integrating intra-tumoral, peritumoral (8mm), and clinical US radiomics features enables highly accurate non-invasive differentiation of FA and PT and stratification of PT subtypes. This approach reduces diagnostic ambiguity in Bi-RADS 4 lesions and decreases unnecessary biopsies, demonstrating significant clinical utility for precision diagnosis of breast fibroepithelial tumors.

## 1 Introduction

Accurate differential diagnosis of breast tumors remains a core clinical challenge. Radiomics, through high-throughput extraction of medical imaging features, has established a new paradigm for non-invasive diagnosis (1, 2). Our previous research developed a differentiation model between benign and malignant breast tumors using multimodal deep learning radiomics (including mammography, MRI, and ultrasound), addressing the fundamental classification issue (3). However, for common benign breast tumors such as fibroadenomas (FA) and potentially malignant phyllodes tumors (PT), there exist distinct subtypes with significant differences in biological behavior and prognosis (4, 5). Breast ultrasound plays a crucial role in differentiating between FA and PT (6).

Breast fibroepithelial lesions encompass a heterogeneous group of biphasic tumors, including FA and PT. PTs were first identified in 1838 and described as cystosarcomas phyllodes due to their leaf-like appearance (7, 8). These tumors account for 2.5% of all breast fibroepithelial lesions and represent less than 1% of all breast tumors (9, 10). Notably, PT exhibits higher incidence rates in Asian populations, particularly among young women. Based on histological features (stromal cellularity, atypia, mitotic activity, stromal overgrowth, and margin characteristics), PTs are classified into three subtypes: benign, borderline, and malignant (5, 11). PT typically recurs locally within 2–3 years after diagnosis, with total recurrence rates of 10-17% for benign, 14-25% for borderline, and 23-30% for malignant PTs (12, 13).

Therefore, extensive local resection is required for malignant PT. Metastasis in PT indicates poor prognosis, increased mortality risk, and reduced survival rates. In patients with benign or borderline PT, distant metastasis is extremely rare. The occurrence of distant metastasis in benign or borderline PT is likely due to insufficient sample size and misclassification. Thus, precise tumor grading, particularly accurate diagnosis of malignant PT, becomes crucial. Patients with malignant PT should consider systemic treatments such as chemotherapy or targeted therapy, while those with borderline PT have minimal or no metastatic risk, allowing safe surgical intervention (14). Accurate preoperative diagnosis of FA, PT cell tumors, and adenomas not only aids in formulating precise surgical plans and determining appropriate tumor margins and axillary dissection but also helps avoid overtreatment or under-treatment (15). Currently, histological morphology remains the sole diagnostic criterion for malignant PT. However, since PT themselves represent a continuous disease spectrum with overlapping histological features, and given the numerous subjective evaluation parameters used in current grading systems, pathologists lack consensus in classifying malignant PT, which may not fully align with their biological behavior. Previous studies have shown that older age, rapid growth of lesions, and tumors larger than 3 cm are more likely to become borderline and malignant PT (16).Another study observed that there was about 60% inconsistency between biopsy pathology and resection pathology, which may be attributed to insufficient sample size and tumor heterogeneity (17). Therefore, the existing grading criteria for FA and PT are challenged, and accurate and reproducible grading is worth further exploration.

The characteristics of tumor heterogeneity are hemorrhagic areas, cystic changes, high cell density, necrosis and mucinous changes (18). In recent years, with the development of radiological examination techniques, including breast ultrasound, mammography and magnetic resonance imaging have been widely used in the diagnosis and treatment evaluation of FA and PT of the breast. However, traditional ultrasound imaging demonstrates limited potential in predicting FA and PT, and its classification. At present, tumor cells and tumor microenvironment (Tumor microenvironment, TME) are considered to be the key factors in cancer occurrence and development, as well as potential targets for treatment (19).Tumor cells coexist with a variety of cellular components in the tumor microenvironment, forming a more complex tumor immune microenvironment composed of immune infiltrating cells than normal healthy tissues, which has become a hot spot in the diagnosis and treatment of breast tumors (20).

Building on previous research, we integrate radiomics and deep learning to comprehensively evaluate tumor sites using ultrasound imaging for distinguishing these challenging entities. This study explores the deep information mining capabilities of ultrasound, integrating intra-tumor (ITR) and peritumoral (PTR) data to achieve precise differentiation between FA and PT and subtyping of PT. The novelty of this work lies in: 1) the multicenter design; 2) the combined analysis of ITR, PTR (across multiple expansions), and clinical features; 3) the direct comparison of radiomics and deep learning approaches; and 4) the analysis of potential biopsy reduction.

## 2 Materials and methods

### 2.1 Patient population and study design

This retrospective, multicenter study was reviewed and approved by the Ethics Committee of the General Hospital of the Northern Theater Command (Approval No.: Y (2404)-030). The need for informed consent was waived. Data were extracted from ultrasound imaging and clinical pathology records of three medical centers (General Hospital of the Northern Theater Command, Liaoning Provincial Cancer Hospital, and The Fourth Affiliated Hospital of China Medical University) between January 2018 and May 2024.

**Figure 1:**
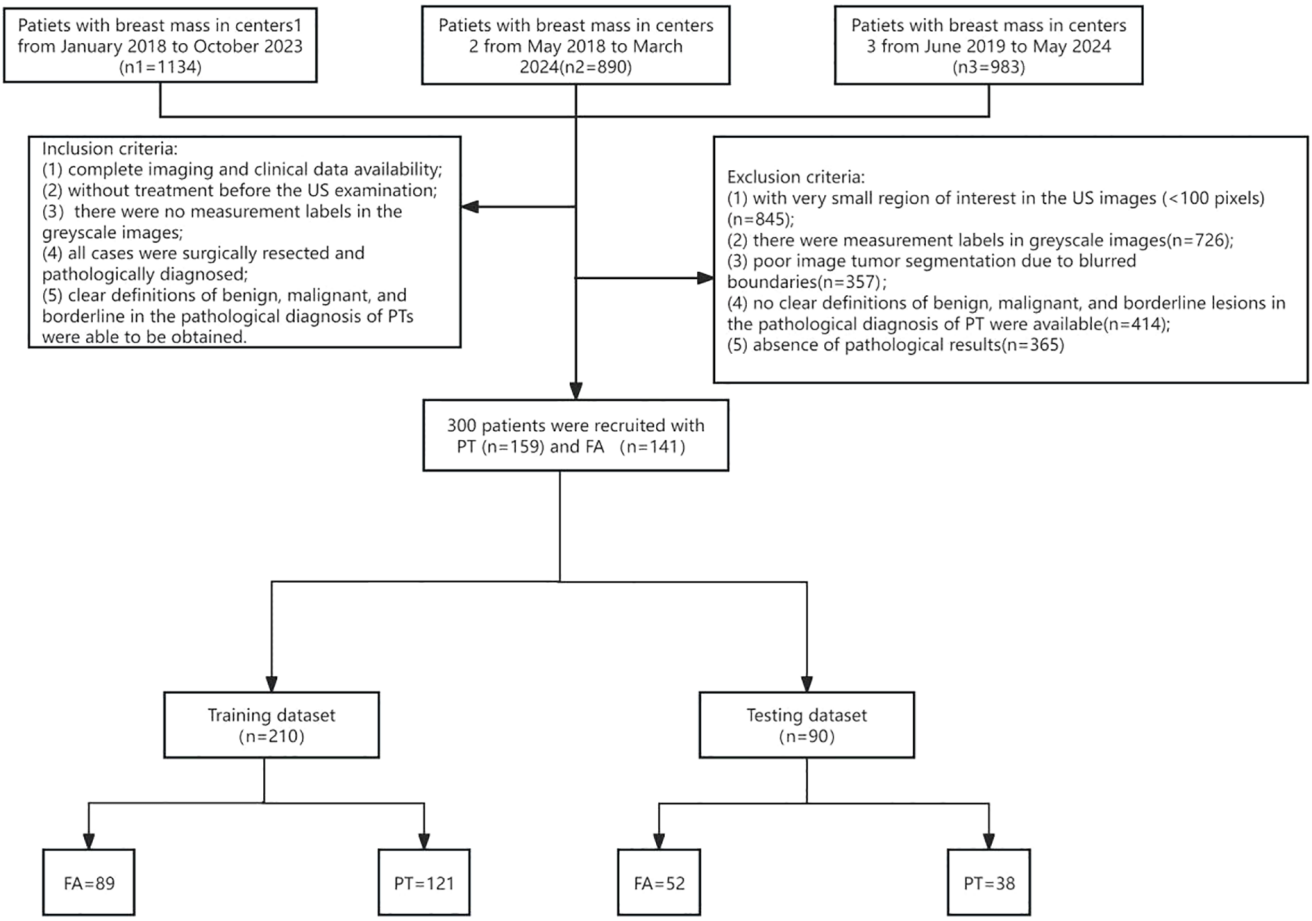
The patient enrollment flowchart.

Inclusion criteria were: (1) Complete availability of imaging and clinical data; (2) No prior treatment before ultrasound examination; (3) No measurement markers in ultrasound grayscale images; (4) All cases underwent biopsy or surgical resection with pathological confirmation; (5) Pathological diagnosis of PT could clearly define subtypes: benign, borderline, and malignant. Exclusion criteria included:(1) Small ROI (<100 pixels); (2) Measurement markers in images; (3) Poor tumor segmentation due to blurred borders; (4) Unclear pathological diagnosis; (5) Lack of definitive pathological results. Based on these criteria, 300 female breast tumor patients were enrolled: 141 with FA and 159 with PT. The patient enrollment flowchart is shown in **Figure 1**.

### 2.2 Ultrasound image acquisition and preprocessing

Breast ultrasound examinations were performed by specialists using systems(Mindray DC-7, GE Logiq E9, GE Voluson E8, Philips IU22, GE Logiq E20) equipped with 3–13 MHz linear array transducers. Examination followed ACR BI-RADS 5th edition guidelines (21). Scanning parameters (depth: 4–5 cm; gain: 10–25 dB; dynamic range: 70 dB; frame rate: 26 fps) were adjusted per patient based on habitus and lesion location, following standard clinical protocols at each center. Patients were positioned supine or lateral. The focal area was centered on the lesion. Images were stored in PACS. For each lesion, a specialist selected five representative images (longest axis, perpendicular, three other clear angles).

**Figure 2:**
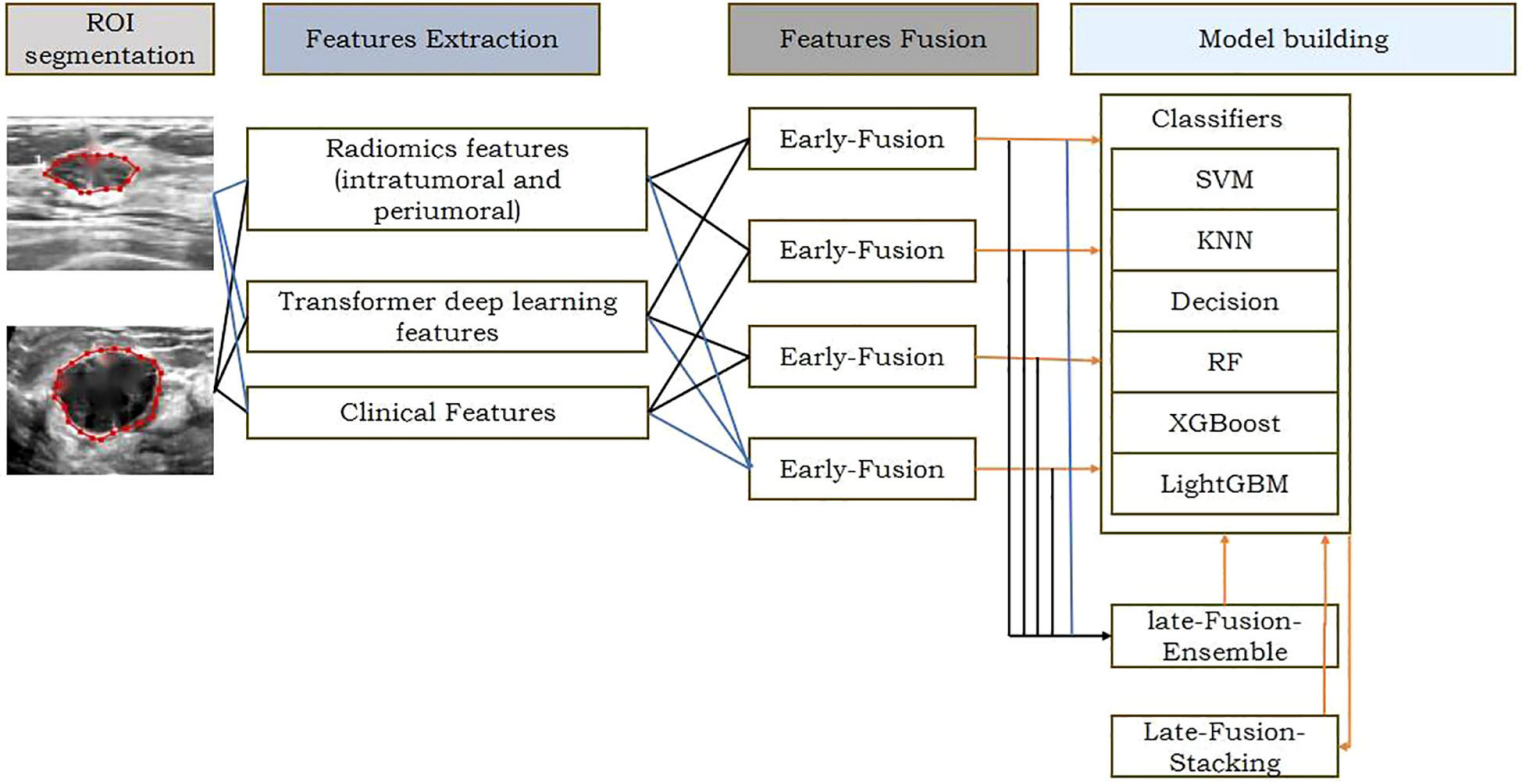
The workflow of ROl extraction and preprocessin DLR: conventional radiomic features were extracted from US datas. Feature selection and fusion techniques were applied to reduce dimensionality and integrate complementary information. Classification modeling was done using 6 machine learning algorithms.

**Figure 3:**
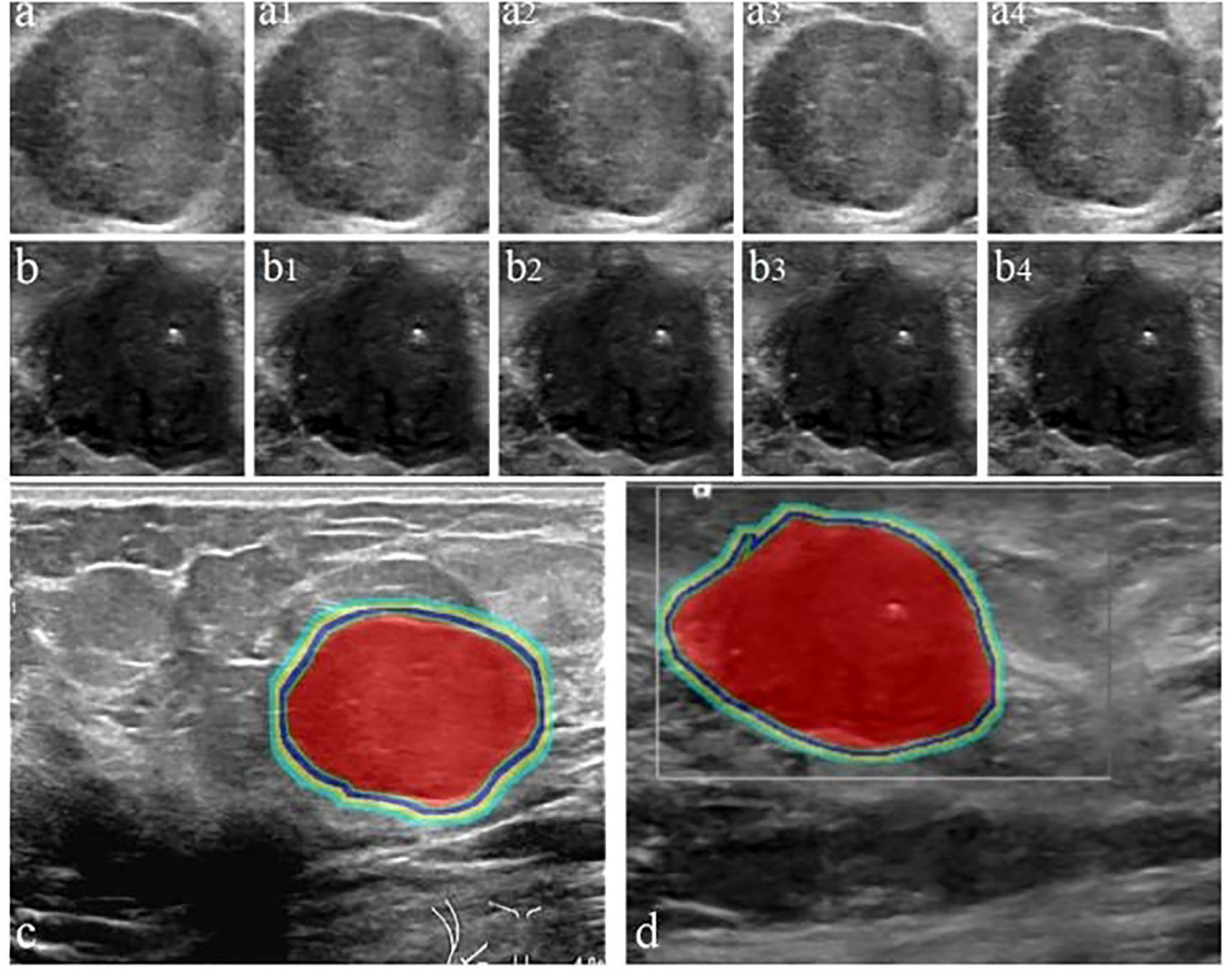
Workflow for delineating ITR and PTR in FA and PT patients under different ROI segmentation schemes: FA **(a)** and PT **(b)**; red areas represent ITR, yellow-green areas represent 4 mm, 8 mm, 12 mm, and 16 mm PTR in FA **(c)** and PT **(d)**. ITR: intratumoral area; PTR: peritumoral area.

**Figure 4:**
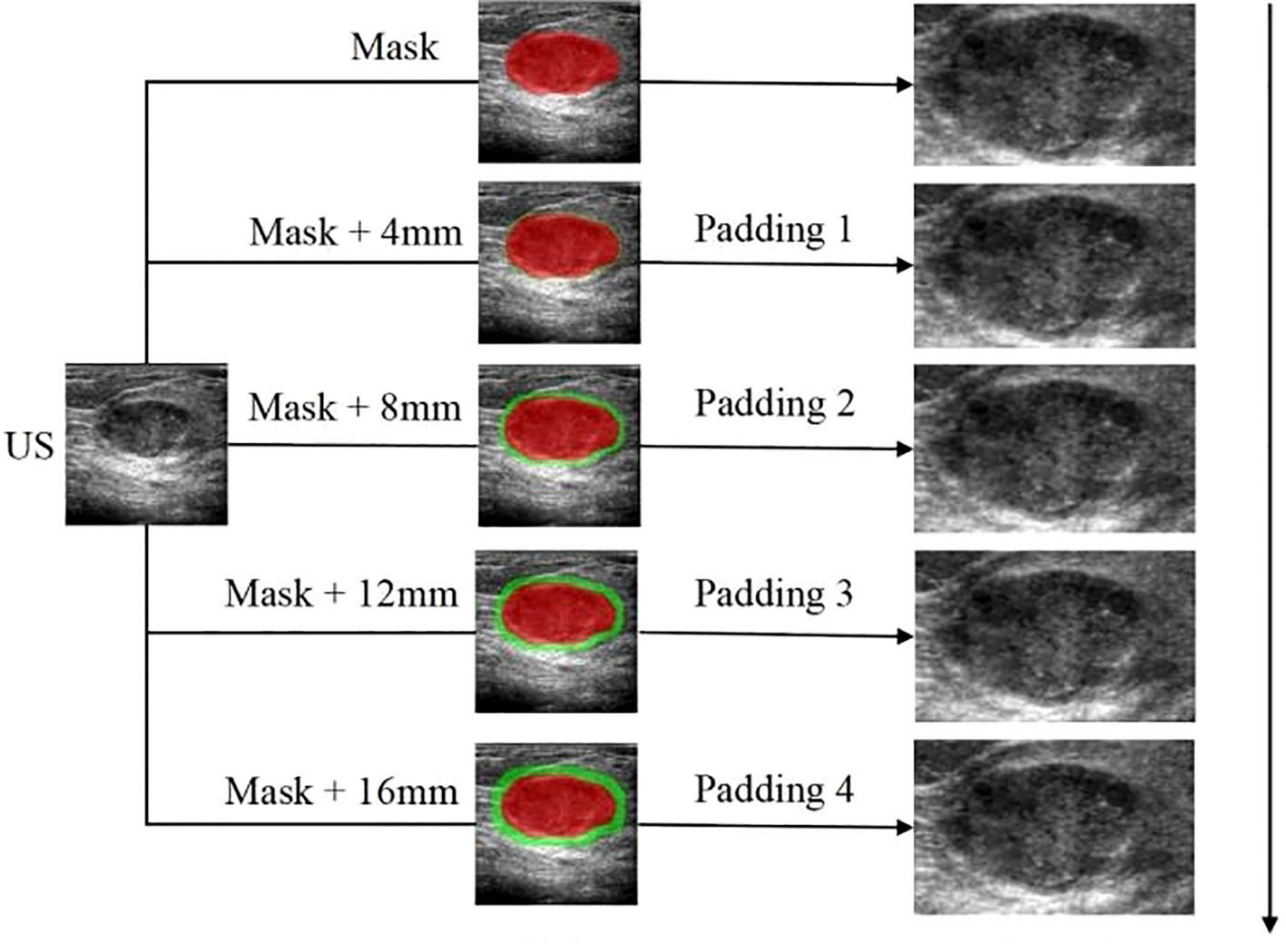
The workflow of the radiomics analysis of ITR and PTR:The ROI delineation of the tumor was increased by 4mm layer by layer to 16mm; The peritumoral lesion image gradually increased by 4mm to 16mm.

ROI delineation and image segmentation were performed manually using ITK-SNAP (v3.80) by three ultrasound specialists with >10 years of experience, guided by a senior physician (>20 years experience). Inter- and intra-observer variability was assessed using Dice Similarity Coefficient (DSC) and Intraclass Correlation Coefficient (ICC); results indicated good to excellent agreement (DSC: 0.78-0.93, ICC: 0.832-0.949), and no statistically significant bias was found in feature extraction between observers (p > 0.05 for most features). The ITR was delineated layer-by-layer, avoiding necrosis. PTR was generated by expanding the ITR outward by 4mm, 8mm, 12mm, and 16mm using the Onekey AI platform (22) and MATLAB 2016b (23). The rationale for selecting these PTR thicknesses was based on prior literature suggesting stromal involvement within these distances (24, 25) and exploratory analysis showing varying predictive power across these ranges (see Sensitivity Analysis in Section 2.6). PTR extending beyond breast parenchyma was manually removed. Disagreements were resolved by consensus. Examples are shown in **Figures 2**–**4**.

### 2.3 Feature extraction

Using PyRadiomics (v3.0.1) (26), 114 radiomics features were extracted per ROI: 18 first-order statistics, 14 shape-based, 75 textural features (GLCM, GLRLM, GLSZM, GLDM, NGTDM) (21, 27). Feature extraction settings were default PyRadiomics parameters unless otherwise specified (binWidth: 25; force2D: True).

### 2.4 Feature selection and model construction

High-dimensional features underwent dimensionality reduction to prevent overfitting. Z-score normalization was applied. The Boruta algorithm (22) was used for feature selection, comparing original features against shadow features. LASSO regression was also employed for optimal feature subset selection.

A two-stage classification model was developed: 1) Diagnostic network differentiating FA from PT using ITR, PTR, and combined features; 2) Grading network classifying PT into benign, borderline, malignant. The models were constructed using six machine learning classifiers: Random Forest (RF; n_estimators=100), Support Vector Machine (SVM; kernel=‘rbf’, C = 1.0), XGBoost (XGB; max_depth=6, learning_rate=0.1), LightGBM (LGBM; num_leaves=31), Decision Tree (DT; max_depth=5), and Logistic Regression (LR; solver=‘liblinear’). Hyperparameters were optimized using 5-fold cross-validation GridSearch within the training set. Five deep learning models [CNN (28), MLP (29), ViT (17), GAN (30), RNN (31)] were also implemented using standard architectures and trained from scratch using Adam optimizer (learning_rate=0.001), batch size=16, for 100 epochs with early stopping.

### 2.5 Data split and validation strategy

To mitigate the risk of data leakage, patient-level splitting was strictly enforced. The dataset was first stratified by center and pathology label (FA, PT benign, PT borderline, PT malignant). Patients were then randomly split 7:3 into training (210 patients) and internal testing (90 patients) sets, ensuring no patient’s images appeared in both sets. For external validation, each center’s data was held out iteratively: models trained on data from two centers were tested on the third center’s data. This per-center hold-out test provides a robust estimate of generalizability. The sample size of 300 patients was based on a pragmatic sample availability from the collaborating centers over the study period. A *post-hoc* power analysis based on the observed AUC (0.96) for the primary outcome (FA vs. PT) indicated sufficient power (>0.95) at alpha=0.05. Performance was assessed via AUC, accuracy, sensitivity, specificity, F1-score. Calibration was evaluated using Hosmer-Lemeshow test and calibration curves. Decision curve analysis (DCA) assessed clinical utility. Five-fold cross-validation was performed on the training set for hyperparameter tuning and internal performance estimation.

### 2.6 Statistical analysis and sensitivity analysis

Statistical analysis used R (v4.3.1) and SPSS (v26.0). Non-normal continuous data summarized as median (IQR); compared using Mann-Whitney U/Kruskal-Wallis tests. Categorical variables as n (%); compared using Chi-square/Fisher’s exact test. Model performance metrics reported with 95% confidence intervals (CI) calculated via bootstrap (2000 repetitions). AUC comparisons used DeLong test. Inter-observer agreement used ICC and Cohen’s Kappa. P<0.05 significant. A sensitivity analysis was performed for the PTR thickness parameter. Models were built and evaluated using only ITR, and ITR combined with each PTR thickness (4, 8, 12, 16 mm). The 8mm PTR expansion yielded the highest average AUC across classifiers for the FA vs. PT task (See **Supplementary Table S1**), justifying its selection as the optimal PTR width for the combined model.

## 3 Results

### 3.1 Clinical data characteristics

Among the 300 breast mass patients enrolled in the study, 141 were ultimately diagnosed with FA and 159 with PT through postoperative pathological examination. A randomized 7:3 split was used to divide patients into a training set (210 cases) and a validation set (90 cases). No significant differences were observed between the training and validation sets in terms of age, lesion diameter, location, menopausal status, clinical symptoms, growth rate, hardness, mobility, margins, echogenicity, blood flow, or BI-RADS classification (all P> 0.05). Each patient had a single lesion in one breast and underwent surgical treatment: 85 patients (28.3%) received breast biopsy, 110 patients (36.7%) underwent local excision, 73 patients (24.3%) had wide excision, and 32 patients (10.6%) underwent radical mastectomy. Detailed demographic data and lesion characteristics are summarized in **Table 1**.

**Table 1:** The baseline information.

Items	Training set (n=210)				Test set (n=90)			
	FA(n=89)	PT(n=121)	Value	P	FA(n=38)	PT(n=52)	Value	P
Case	89(42.4%)	121(57.6%)			38(42.2%)	52(57.8%)	0.001	0.980
Images	280	395			123	165	0.011	0.724
Age	48.03+1.29	50.20+1.77	0.829	0.408	57.16+2.47	51.13+2.44	1.696	0.093
Diameter	3.22+0.14	3.52+0.13	1.592	0.112	3.75+0.24	3.12+0.21	1.964	0.053
Position*							6.027	0.925
Centre	3(3.4%)	3(2.5%)			1(2.6%)	1(1.9%)		
UIQ	21(23.6%)	32(26.4%)			10(26.3%)	16(30.8%)		
LIQ	24(27.0%)	33(27.3%)			6(15.8%)	12(23.1%)		
LOQ	22(24.7%)	26(21.5%)			11(28.9%)	8(15.4%)		
UOQ	19(21.3%)	27(22.3%)			10(26.3%)	15(28.8%)		
Menopause							1.364	0.714
Yes	51(57.3%)	63(52.1%)			18(47.4%)	26(50.0%)		
No	38(42.7%)	58(47.9%)			20(52.6%)	26(50.0%)		
Clinlcal symptom							1.946	0.583
Positive	43(48.3%)	56(46.3%)			14(36.8%)	21(40.4%)		
Negative	46(51.7%)	65(53.7%)			24(63.2%)	31(59.6%)		
Growth							1.713	0.634
Fast	40(44.9%)	64(52.9%)			17(44.7%)	24(46.2%)		
Slow	49(55.1%)	57(47.1%)			21(55.3%)	28(53.8%)		
Hardness							12.187	0.051
Soft	42(47.2%)	38(31.4%)			13(34.2%)	20(38.5%)		
Middle	25(28.1%)	36(29.8%)			17(44.7%)	20(38.5%)		
Hard	22(24.7%)	47(38.8%)			8(21.1%)	12(23.0)		
Activity							0.675	0.879
Good	50(56.2%)	63(52.1%)			22(57.9%)	27(51.9%)		
Bad	39(43.8%)	58(47.9%)			16(42.1%)	25(48.1%)		
Border							3.280	0.350
Clear	64(71.9%)	84(69.4%)			26(68.4%)	30(57.7%)		
Irregular	25(28.1%)	37(30.6%)			12(31.6%)	22(42.3%)		
Echo							2.944	0.400
Even	45(50.6%)	75(62.0%)			23(60.5%)	29(55.8%)		
Inhomogeneous	44(49.4%)	46(38.0%)			15(39.5%)	23(44.2%)		
Blood flow							2.459	0.483
Yes	42(47.2%)	54(44.6%)			18(47.4%)	18(34.6%)		
No	47(52.8%)	67(55.4%)			20(52.6%)	34(65.4%)		
BiRADS							11.967	0.448
3	36(40.4%)	32(26.4%)			8(21.1%)	10(19.2%)		
4a	22(24.7%)	29(24.0%)			9(23.7%)	16(30.8%)		
4b	15(16.9%)	31(25.6%)			10(26.3%)	12(23.1%)		
4c	10(11.2%)	19(15.7%)			8(21.1%)	10(19.2%)		
5	6(6.7%)	10(8.3%)			3(7.9%)	4(7.7%)		

*FA, Fibroadenoma; PT, Phyllodes Tumor; UOQ, upper outer quadrant; UIQ, upper inner quadrant; LIQ, lower inner quadrant; LOQ, lower outer quadrant.

### 3.2 Feature consistency and selection

Dice coefficients for ROI segmentation were 0.78-0.92,0.80-0.93, 0.84-0.92 for the three physicians. ICCs were 0.832-0.949 (intra-observer) and 0.742-0.925 (inter-observer), indicating good reproducibility. LASSO selected 5 optimal features from ITR and 10 from 8mm PTR(1 first-order, 9 higher-order).

### 3.3 Predictive performance of the imaging radiomics model

Performance metrics for Models 1–7 are detailed in **Table 2**. The combined Model 7 (ITR + 8mm PTR+Clinical) performed best for FA/PT differentiation (AUC: 0.960, Accuracy: 96.0%, Sensitivity: 96.0%, Specificity: 94.5%). For PT subtyping, Model 7 (Light GBM classifier) achieved an AUC of 0.874 (95% CI: 0.798-0.950), Accuracy: 77.2%.

**Table 2:** Results of radiomic classification utilizing intratumoral and peritumoral information of US imaging.

Model	Accuracy	AUC	Sensitivity	Specificity	Accuracy	Recall	F1	Classifier
1(ITR)	0.848	0.935	0.867	0.862	0.913	0.840	0.875	XG Boost
2(PTR4)	0.550	0.573	0.467	0.800	0.875	0.467	0.609	Light GBM
3(PTR8)	0.750	0.747	0.733	0.800	0.917	0.733	0.815	RF
4(PTR12)	0.450	0.400	0.333	0.800	0.833	0.333	0.476	SVM
5(PTR16)	0.550	0.360	0.600	0.400	0.750	0.600	0.667	RF
6(ITR+PTR8)	0.894	0.958	0.906	0.931	0.945	0.932	0.927	XGB
7(ITR+PTR8+clinical)	0.960	0.960	0.960	0.945	0.971	0.960	0.965	LGBM

*ITR, Intratumoral Region; PTR, Peritumoral Region; XGB, eXtreme Gradient Boosting; LGBM, Light Gradient Boosting Machine; RF, Random Forest; SVM, Support Vector Machine

### 3.4 Predictive performance of deep learning models

Performance of DL models is shown in **Table 3**. GAN performed best for FA/PT (AUC: 0.976). MLP performed best for PT subtyping (AUC: 0.950). The relatively lower performance of CNN and ViT compared to GAN might be attributed to the GAN’s potential to learn more robust feature representations from limited data through its adversarial training paradigm, whereas standard CNNs and ViTs may require larger datasets to achieve optimal performance in this specific task.

**Table 3:** Results of deep learning information of US imaging.

Model	Accuracy	AUC	Sensitivity	Specificity	Accuracy	Recall	F1
CNN	0.531	0.498	0.421	0.692	0.667	0.421	0.516
MLP	0.746	0.944	0.630	0.577	0.577	0.789	0.667
ViT	0.625	0.401	0.947	0.154	0.621	0.947	0.750
GAN	0.896	0.976	0.870	0.952	0.976	0.870	0.920
RNN	0.750	0.733	0.733	0.800	0.917	0.733	0.815

*CNN, Convolutional Neural Network; MLP, Multilayer Perceptron; ViT, Vision Transformer; GAN, Generative Adversarial Network; RNN, Recurrent Neural Network.

### 3.5 Subgroup analysis based on BI-RADS

To evaluate the impact of BI-RADS classification, patients were divided into three subgroups: Grade 3 (n=86,42 PT cases), Grade 4 (including Grade 4a [76 cases], Grade 4b [68 cases], and Grade 4c [47 cases]), and Grade 5 (n=23,14 PT cases). In all three subgroups (n=191, 117 PT cases and n=23,14 PT cases), Model 7 (ITR+PTR8+clinical) showed high diagnostic accuracy across BI-RADS subgroups: Grade 3: 84.0%, Grade 4: 66.0%, Grade 5: 88.0%. Specificity was highest for Grade 5 lesions (96.6%). Notably, in the Grade 4 lesion subgroup, Model 7 showed enhanced specificity when incorporating intratumoral and peritumoral (8mm) clinical features. This highlights the potential of integrating tumor-intrusion, peritumoral, and clinical characteristics for addressing diagnostic challenges in BI-RADS Grade 4 lesions.

### 3.6 Biopsy reduction analysis

Model 7 achieved an overall biopsy reduction rate of 11.7% (10/85), with an 18.1% (6/33) reduction for PT cases. Confidence intervals (95%) for the reduction rates were calculated using the Clopper-Pearson exact method: Overall: 11.7% (5.7%-20.6%); PT: 18.1% (7.0%-35.5%). Results are in **Table 4**.

**Table 4:** Performance of different models in reducing the rate of lesion biopsy.

Model	Biopsy reduction rate	FA	PT	Values	P value
1(TR)	5.8% (5/85)	5.7% (3/52)	6.1% (2/33)	0.035	0.852
6(ITR+PTR8)	8.2%(7/85)	7.7% (4/52)	9.0% (3/33)	0.004	0.949
7(ITR+PTR8+clinical)	11.7%(5.7%-20.6%)	7.7% (5.5%-14.8%)	18.1%(7.0%-35.5%)	1.191	0.275

## 4 Discussion

Compared with traditional visual-based image evaluation methods, radiological features derived from imaging can uncover additional latent characteristics. This radiomics-based approach demonstrated potential in distinguishing between FA and PT. Some findings revealed that the optimal combination of imaging radiomics features included high width-to-height ratio, edge blurriness, machine learning, energy, gray entropy, and intramural calcification could achieve good performance. In this study, we developed a model using radiomics features extracted from 114 breast lesions within each ROI through grayscale co-occurrence matrix (GLCM), grayscale run-length matrix (GLRLM), grayscale size zone matrix (GLSZM), grayscale-dependent matrix (GLDM), and neighborhood grayscale difference matrix (NGTDM). The diagnostic efficacy metrics for FA and PT showed accuracy, AUC, sensitivity, specificity, precision, recall, and F1 values of 84.8%, 0.935, 86.7%, 86.2%, 91.3%, and 84.0% respectively. Our model demonstrated slightly higher accuracy than some studies (17, 30), likely due to the distinct radiomics feature extraction. Domestic research indicates that AI-assisted diagnosis through radiomics and deep learning can identify subtle morphological and textural differences between PT and FA, thereby enhancing diagnostic efficacy.

Previous radiomics analyses primarily focused on visual tumor boundaries, but recent studies have also emphasized peritumoral regional information. Researchers propose that the peritumoral area may be the earliest site of tissue lesion development and plays a critical role in determining tumor progression and treatment response (32). However, inflammatory tissues (characterizing the transition between tumors and normal parenchyma) introduce certain misleading factors into radiographic results. Therefore, clinical visual analysis alone often fails to detect peritumoral regions, whose extent may influence tumor diversity (33). Given the varying complexities of different diseases and pathological issues, standardized end-to-end solutions are typically unavailable for comprehensive analysis. Instead, parameterized approaches tailored to specific tumor locations and imaging modalities are often required. Zhang (34)developed a linear discriminant analysis (LDA) model integrating three radiomics features (from ITR, 5mm PTR, and ITR + 10mm PTR) with two clinical factors (age and BI-RADS classification), demonstrating strong predictive power in both internal and external test datasets. This model, which combines intratumoral and peritumoral radiomics features with clinical factors, successfully predicted malignant BI-RADS 4 lesions in contrast-enhanced mammography using AUC values of 0.907 and 0.904 respectively. Our study demonstrates that integrating ITR, PTR (8mm), and clinical features achieves excellent performance in differentiating FA from PT and grading PT subtypes using US radiomics. The optimal 8mm PTR likely captures critical stromal alterations and tumor-host interactions at the invasive edge, a known hotspot for biological activity in breast tumors (35, 36).

In our model 7, which integrates intratumoral +8mmPTR with clinical features (diameter and BI-RADS classification), we observed slightly lower sensitivity and accuracy compared to the previous model in FA and PT cases classified as BI-RADS 4. However, it demonstrated higher specificity. These differences may stem from subtle variations in the microscopic structures of FA and PT within the selected multicenter ultrasound imaging data. Huang (37)emphasized not only clinically-derived models but also the importance of comprehensive radiomics analysis for accurate breast nodule characterization. Our findings reveal that the combined model integrating ITR + 8mm PTR+clinical features achieves optimal diagnostic precision. These results demonstrate that ultrasound-based intratumoral/peritumoral features combined with clinical characteristics exhibit superior diagnostic efficacy in both FA/PT classification models and pathological subtypes grading models for PT, thereby enhancing diagnostic accuracy and supporting clinical decision-making. Our model significantly reduced the potential need for biopsies, especially for PT lesions. This aligns with the goal of precision medicine to minimize invasive procedures (32). And potential clinical integration could involve PACS-integrated software for automatic feature extraction and model inference, providing real-time decision support during ultrasound examination and potentially reducing workflow interruptions.

This study also has some limitations. A key limitation is the retrospective design and class imbalance, particularly for borderline and malignant PT subtypes, which may introduce selection bias and affect model generalizability. Future prospective studies with larger, balanced cohorts are needed. Furthermore, this study utilized only ultrasound. While US is crucial, incorporating multimodal imaging (mammography, MRI) in future work could potentially enhance performance further.

## 5 Conclusion

This study established ultrasound-based intra-tumoral, peritumoral, and clinical radiomics features. Diagnostic efficacy for FA and PT was first evaluated. Building on this foundation, further classified PT into benign, borderline, and malignant subtypes, and analyzed performance across different BI-RADS grades, and identified ITR and PTR characteristics associated with reduced biopsy rates. In conclusion, the proposed US-based radiomics model integrating intra-tumoral, peritumoral (8mm), and clinical features serves as an effective non-invasive tool for differentiating FA from PT and classifying PT subtypes. It shows particular value in managing BI-RADS 4 lesions and reducing unnecessary biopsies. Future work should focus on large-scale, prospective, multicenter validation and exploration of multimodal integration.

## Data Availability

The datas in this article would be apply to the authors. Requests to access the datasets should be directed to guoxiu_16@sina.com.
