# Supplementary material for: Intra-tumor and peritumoral radiomics and deep learning based on ultrasound for differentiating fibroadenoma and phyllodes tumor: a multicenter study
Source: Front Oncol. 2025 Oct 23;15:1668793. doi: 10.3389/fonc.2025.1668793 (PMC12588848; doi:10.3389/fonc.2025.1668793)
Supplement: Supplementary file 1 [file Table1.docx]

Supplementary Table S1 The PTR thickness classifiers for the FA and PT task

| Model | accuracy | AUC | sensitivity | specificity | accuracy | recall | F1 | classifier |
| --- | --- | --- | --- | --- | --- | --- | --- | --- |
| 4mm (PTR4) | 0.550 | 0.573 | 0.467 | 0.800 | 0.875 | 0.467 | 0.609 | Light GBM |
| 8mm (PTR8) | 0.750 | 0.747 | 0.733 | 0.800 | 0.917 | 0.733 | 0.815 | RF |
| 12mm (PTR12) | 0.450 | 0.400 | 0.333 | 0.800 | 0.833 | 0.333 | 0.476 | SVM |
| 16mm (PTR16) | 0.550 | 0.360 | 0.600 | 0.400 | 0.750 | 0.600 | 0.667 | RF |

*ITR：Intratumoral Region；PTR：Peritumoral Region；Light GBM： Light Gradient Boosting Machine;RF:Random Forest;SVM:Support Vector Machine.
